# From type 1 diabetes biology to therapy: The Human Islet Research Network

**DOI:** 10.1016/j.molmet.2021.101283

**Published:** 2021-07-03

**Authors:** John S. Kaddis, Layla Rouse, Audrey V. Parent, Diane C. Saunders, Anath Shalev, Cherie L. Stabler, Doris A. Stoffers, Bridget K. Wagner, Joyce C. Niland

**Affiliations:** 1 Department of Diabetes and Cancer Discovery Science, Arthur Riggs Diabetes and Metabolism Research Institute, Beckman Research Institute, City of Hope, Duarte, CA, USA; 2 Diabetes Center, Department of Medicine, University of California, San Francisco, San Francisco, CA, USA; 3 Division of Diabetes, Endocrinology and Metabolism, Department of Medicine, Vanderbilt University Medical Center, Nashville, TN, USA; 4 Comprehensive Diabetes Center, Department of Medicine, Division of Endocrinology, Diabetes and Metabolism, University of Alabama at Birmingham, Birmingham, AL, USA; 5 J. Crayton Pruitt Family, Department of Biomedical Engineering, Herbert Wertheim College of Engineering, University of Florida, Gainesville, FL, USA; 6 University of Florida Diabetes Institute, University of Florida, Gainesville, FL, USA; 7 Institute for Diabetes, Obesity, and Metabolism and Department of Medicine, Perelman School of Medicine at the University of Pennsylvania, Philadelphia, PA, USA; 8 Chemical Biology and Therapeutics Science Program, Broad Institute, Cambridge, MA, USA

**Keywords:** Type 1 Diabetes, Human Islet, Beta-cell biology, Immunology, CBDS, Consortium on Beta Cell Death and Survival, CHIB, Consortium on Human Islet Biomimetics, CMAI, Consortium on Modeling Autoimmune Interactions, CTAR, Consortium on Targeting and Regeneration, HIREC, Human Islet Research Enhancement Center, HIRN, Human Islet Research Network, HPAC, Human Pancreas Analysis Consortium, HPAP, Human Pancreas Analysis Program, T1D, Type 1 Diabetes

The last 20 years has been a remarkable period in the history of diabetes research. For the first time, pancreata, Islets of Langerhans, relevant tissue, and blood samples from humans have become more widely accessible to investigators around the world [1, 2, 3, [4], 5]. The convergence of these resources has energized a generation of research and investigators that are delivering new insights into long-standing questions in diabetes.

Recognizing this opportunity, in 2014 the National Institute of Diabetes and Digestive and Kidney Diseases (NIDDK) established the Human Islet Research Network (HIRN) to conduct innovative and collaborative research investigating the loss of human beta cells in type 1 diabetes (T1D), and to identify pioneering strategies to protect and replace functional beta-cell mass. The HIRN is organized as a modular network of distinct research consortia, each concentrated on common biological or technical questions and challenges in T1D. This design offers flexibility to respond to emerging opportunities and the latest scientific discoveries, as well as a means of sharing and collaborating across and within consortia. A Human Islet Research Enhancement Center (HIREC) provides support to facilitate effective group collaboration, and informatics tools to meet the challenges of working in a team science environment. New innovative science is recruited to the HIRN through competitive funding opportunities open to all scientists, regardless of HIRN affiliation. Expert scientific panel members, independent of the HIRN, provide objective feedback and guidance to the Network and each consortium. HIRN interactions, opportunities, shared visions, and goals are promoted by a Trans-Network Committee, comprised of NIDDK staff, representatives from each consortium steering committee, and the HIREC. Promoting the careers of junior investigators and encouraging more scientists to pursue T1D research are high priorities of HIRN. In all, this advanced team science initiative brings together over 280 investigators and trainees across more than 70 peer-reviewed grants.

The HIRN is comprised of five consortia (Figure 1). The Consortium on Modeling Autoimmune Interactions (CMAI) focuses on studies of human immunity and pancreatic islets, with the goals of developing tools, reagents, and resources to build robust models of the human immune system to help unravel the T1D auto-immune process and reverse or stop this destruction. The Consortium on Beta Cell Death and Survival (CBDS) studies the “silent” disease period of T1D prior to diagnosis; while challenging to evaluate, this period in which disease develops may hold important clues as to why and how beta cells die in T1D, and provide a window of opportunity to arrest this process before T1D develops. The Consortium on Targeting and Regeneration (CTAR) investigates new approaches toward the safe and controlled replenishment of functional beta cells, potentially freeing patients from daily insulin therapy, and methods to specifically target the islet milieu with new therapeutics. The Consortium on Human Islet Biomimetics (CHIB) engineers in vitro platforms capable of supporting the long-term, dynamic investigation and interrogation of islets and immune cells on the benchtop. The Human Pancreas Analysis Consortium (HPAC) investigates the physical and functional organization of the human islet tissue environment and the cell–cell relationships within the pancreatic tissue ecosystem. It includes a resource-generation component, the Human Pancreas Analysis Program (HPAP), that is performing deep phenotyping of the human endocrine pancreas and its interaction with the immune system, to better understand the cellular and molecular events that precede and lead to the beta cell loss in T1D; a set of independent research projects within HPAC explore the biological contributions of specific pancreatic cell populations to islet cell function and dysfunction via HPAP data. Across all five consortia, the HIREC offers comprehensive administrative, outreach, and scientific coordination, along with the curation of scientific data and resources to facilitate HIRN research. HIRN research is further enabled by key partnerships with the Integrated Islet Distribution Program (IIDP), the Network for Pancreatic Organ Donors with Diabetes (nPOD) initiative, and the NIDDK Information Network (dkNET).

**Figure 1:**
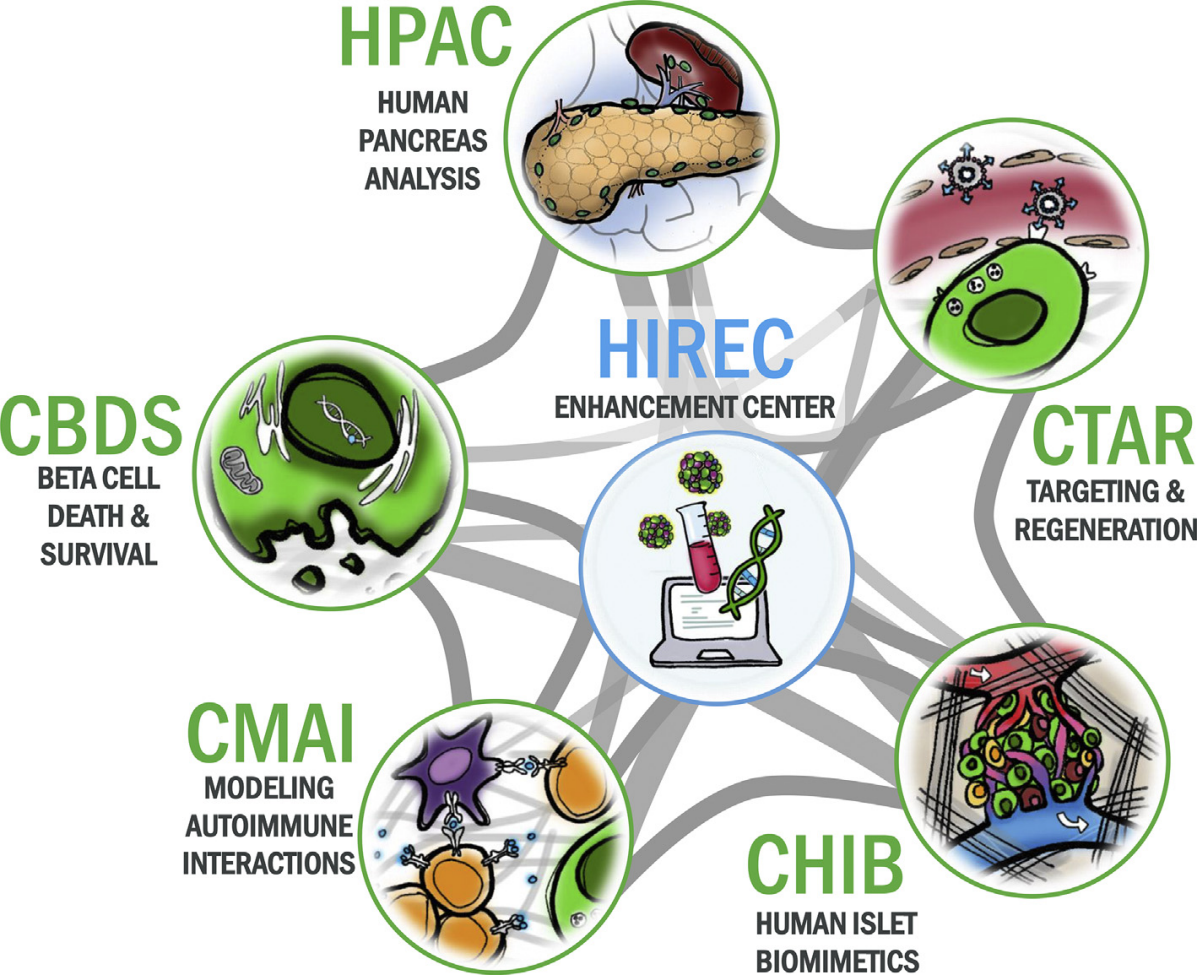
**Overview of the Human Islet Research Network**. The HIRN has been structured to allow scientists to focus on unifying themes and obstacles in type 1 diabetes, while providing a cross-disciplinary collaborative environment for sharing, to advance discoveries and promote therapeutic approaches. This network includes the Human Pancreas and Analysis Consortium (HPAC), the Consortium on Targeting and Regeneration (CTAR), the Consortium on Human Islet Biomimetics (CHIB), the Consortium on Modeling Autoimmune Interactions (CMAI), the Consortium on Beta Cell Death and Survival (CBDS), and the Human Islet Research Network Enhancement Center (HIREC).

In this series of reviews, major advances, questions, and challenges in the field of T1D will be identified by scientists working within or affiliated with the HIRN. Each review provides the historical context of a topic, summarizes the latest breakthroughs in the field, explores how evolving concepts and data are driving new lines of inquiry, and presents grand challenges and future directions.

As pancreatic beta cells play a crucial role in T1D, the series begins by summarizing their key features and presenting criteria to identify them. From there, the series documents the complexities of the beta cell lifecycle, from the diversity of stress it encounters to the interactions and communications it has with its environment. New approaches are then highlighted that address these challenges by modeling and modulating immune mediated responses and beta cell renewal. Finally, innovative biologics and technologies are described which are being developed to discover and deliver the next generation of therapeutics. The reviews in this series exemplify the significant progress the field has made in understanding T1D, and illustrates the potential of these scientific and technological advances.

## Ethics approval and consent to participate

Not applicable.

## Consent for publication

Not applicable.

## Availability of data and materials

Not applicable.

## Funding

This work was written using resources and/or funding provided by the NIDDK-supported Human Islet Research Network (HIRN, HIRN, RRID: SCR_014393; https://hirnetwork.org), including U24DK104162 to JSK, LR, and JCN, U01DK123559 to AVP, U01DK120379 to AS, UG3DK122638 to CLS, UC4DK112217 to DAS, and U01DK123717 to BKW.

## Authors' contributions

JSK, LR, AVP, AS, CLS, DAS, BKW, and JCN contributed to the conception of this work. JSK, LR, and JCN drafted and substantively revised the manuscript. AVP, DCS, AS, CLS, DAS, and BKW substantively revised the manuscript. DCS created the figure. All co-authors approved the submitted version, agree to be personally accountable for his/her contributions, and will ensure that any accuracy or integrity questions are investigated and resolved.
